# Biological monitoring of pesticide exposures in residents living near agricultural land

**DOI:** 10.1186/1471-2458-11-856

**Published:** 2011-11-10

**Authors:** Karen S Galea, Laura MacCalman, Kate Jones, John Cocker, Paul Teedon, Anne J Sleeuwenhoek, John W Cherrie, Martie van Tongeren

**Affiliations:** 1 Institute of Occupational Medicine, Riccarton, Edinburgh, UK; 2 Health and Safety Laboratory, Buxton, UK; 3 School of Engineering and the Built Environment, Glasgow Caledonian University, Glasgow, UK

## Abstract

**Background:**

There is currently a lack of reliable information on the exposures of residents and bystanders to pesticides in the UK. Previous research has shown that the methods currently used for assessing pesticide exposure for regulatory purposes are appropriate for farm workers [1]. However, there were indications that the exposures of bystanders may sometimes be underestimated. The previous study did not collect data for residents. Therefore, this study aims to collect measurements to determine if the current methods and tools are appropriate for assessing pesticide exposure for residents living near agricultural fields.

**Methods/design:**

The study will recruit owners of farms and orchards (hereafter both will be referred to as farms) that spray their agricultural crops with certain specified pesticides, and which have residential areas in close proximity to these fields. Recruited farms will be asked to provide details of their pesticide usage throughout the spray season. Informed consenting residents (adults (18 years and over) and children (aged 4-12 years)) will be asked to provide urine samples and accompanying activity diaries during the spraying season and in addition for a limited number of weeks before/after the spray season to allow background pesticide metabolite levels to be determined. Selected urine samples will be analysed for the pesticide metabolites of interest. Statistical analysis and mathematical modelling will use the laboratory results, along with the additional data collected from the farmers and residents, to determine systemic exposure levels amongst residents. Surveys will be carried out in selected areas of the United Kingdom over two years (2011 and 2012), covering two spraying seasons and the time between the spraying seasons.

**Discussion:**

The described study protocol was implemented for the sample and data collection procedures carried out in 2011. Based on experience to date, no major changes to the protocol are anticipated for the 2012 spray season although the pesticides and regional areas for inclusion in 2012 are still to be confirmed.

## Background

The use of pesticides and their possible health effects is a subject that gives rise to much public concern and discussion, with some people living next to agricultural land in the UK, rightly or wrongly, attributing health problems to their exposure to pesticides sprayed on these fields. The Royal Commission on Environmental Pollution (RCEP) published a report on "Crop Spraying and the Health of Residents and Bystanders" which generated considerable interest and comment [2]. RCEP focussed on the possible risks for chronic fatigue syndrome and multiple chemical sensitivity from bystander or residential exposure. They recognised that the epidemiological literature showing associations between these conditions and pesticide exposure was equivocal, but concluded that the risk between pesticide exposure and chronic health conditions was plausible. This report and the responses to it [3-5] have ensured that the issue has remained in the public eye.

In Great Britain the use of pesticides in agriculture, horticulture, forestry, food storage and the home or garden is regulated to protect human health and the environment. The regulatory system is administered for the Department of Environment Food and Rural Affairs (DEFRA) by the Chemical Regulation Directorate (CRD) of the Health and Safety Executive. The scientific paradigm underpinning the approval of pesticides involves the comparison of estimated human exposure with some limit or limits, below which there is considered to be high confidence that there will be no adverse health effects. The system is generally considered to be conservative such that estimated exposures represent some multiple of the likely exposure. The exposures are typically estimated for those who apply the pesticide, workers who may be involved in post-application activities such as harvesting, and bystanders or residents living nearby. There are no mandated methods to estimate exposure and applicants for pesticide approval may use measurements made during application or other work with the product, other analogous measurement data or one of a number of exposure models, such as POEM (Pesticide Operator Exposure Model).^1 ^In addition the development and validation of a Bystander and Residential Exposure Assessment Model (BREAM) being undertaken by the Silsoe Spray Applications Unit is due for final reporting end 2011 [6].

Biological monitoring of pesticide metabolites in the urine of subjects has shown that the methods currently used in the UK for assessing pesticide exposure for regulatory purposes were likely to be appropriate for farm workers [1]. However, the methods in current use appeared to underestimate the levels of exposure that could occur in bystanders. This study did not collect data for residents. There is currently a lack of reliable exposure information for residents and bystanders in the UK therefore there is a need to carry out further measurements to determine if the current tools and methods are appropriate for assessing exposure for residents living near agricultural fields.

The relatively short biological half-life of modern pesticide compounds or their metabolites in the human body presents a major challenge to linking biological monitoring data to specific spray events. Studies must ensure urine samples are collected within about 24 hours of spraying. Farming activities are inherently unpredictable because of the changing weather and the presence of insects or other potentially damaging infestations. There is generally no communication between farmers and residents about spraying activities and so an individual may not realise that a neighbouring field has been treated with pesticide.

## Aims and objectives

This study aims to investigate pesticide exposure during and outside the spraying season for residents (adults and children) living next to agricultural fields. It also aims to assess whether the exposure models used for pesticide regulatory risk assessment in the UK produce sufficiently conservative estimates to ensure that their health is sufficiently protected. Exposure to pesticides will be measured using urine samples, while a pharmacokinetic model will be used to determine systemic exposure levels amongst residents.

## Methods

### Summary of study design and sample size

Recruitment for the study will concentrate on two groups of people; farmers and residents. Firstly, the study will recruit owners of farms and orchards (hereafter both will be referred to as farms) that spray their agricultural crops with specified pesticides of interest to the study and which have residential areas in close proximity to these fields.

Table 1 provides details of the pesticides being considered in this study. The inclusion of pesticides into the study is restricted to those where validated analytical methods are available to the project team to analyse for the associated urinary metabolites. As further validated methods become available during the study, the list of pesticides for inclusion may be expanded.

**Table 1:** Pesticides of interest to the study

Active Substance	Function	Relevant crops approved^a ^for use
Captan	Fungicide	Apple, pear
Chlormequat	Growth regulator	Cereals
Chlorpyrifos	Insecticide	Apple, cereals, veg incl. potato
Cypermethrin	Insecticide	Apple and various arable crops incl. potato
Deltamethrin	Insecticide	Apple and various arable crops (not potato)
Diquat	Herbicide/desiccant	Various arable crops incl. potato
Iprodione	Fungicide	Field beans, oil seed rape
Penconazole	Fungicide	Apple, blackcurrant, hops
Pririmicarb	Insecticide	Apple and various arable crops incl. potato
Thiophanate-methyl	Fungicide	Wheat, triticale, field beans, oil seed rape

^a^approval in 2011

Recruited farms will be asked to provide details of their planned and actual pesticide usage throughout a spray season.

Residents living within approximately 100 m of the edge of a field belonging to a recruited farm, and that is likely to be treated with pesticides, will be approached to participate in the study. Informed consenting adults (18 years and over) and children in their care (aged 4-12 years) will be asked to complete an initial background questionnaire and then provide urine samples and accompanying activity diaries during the spraying season and also for a limited number of weeks before/after the spraying season to allow background pesticide metabolite levels to be determined.

Collected urine samples selected for laboratory analysis will be analysed by the Health and Safety Laboratory (HSL) for the pesticide metabolites of interest. Statistical analysis and modelling will be undertaken to determine systemic exposure levels amongst residents.

Surveys are planned to be carried out in three areas in the UK (East Lothian, Kent and East Anglia) over two years (2011 and 2012), covering two spraying seasons and the intervals between the spraying seasons. East Lothian and East Anglia are major arable crop growing areas, while most of the orchards in the UK are located in Kent. The method of spraying used for orchards is likely to result in higher exposures and so it is likely that these will represent the worst-case exposure scenario. Sampling in these three areas should result in the collection of exposure data for a variety of agricultural pesticide usage situations.

During the first full survey, to be carried out in East Lothian and Kent, the aim is to recruit 10 farms (6 in East Lothian and 4 in Kent), each with at least 5 participating households with the aim of recruiting 50 adults and approximately 25 children. During the second survey the aim is to recruit 16 farms (6 in East Lothian, 4 in Kent and 6 in East Anglia), again each with 5 participating households, resulting in a study sample of approximately 80 adults and 40 children.

We will compensate each individual participating in the study for their time incurred in completing the questionnaire and providing urine samples by offering them a gift voucher of their choice. A voucher of similar amount will be made available to the parents of the participating children. The value of the voucher is based on a rate of £5 for each questionnaire and urine sample provided, which is a similar rate to that used in a previous study [7] where individuals completed a questionnaire and provided a urine sample. Farmers will be compensated on a similar weekly rate for providing information about spraying events. Participants will be asked to sign a form acknowledging receipt of the compensation.

### Ethical considerations

Full ethical approval for the study has been obtained from the South East Scotland Research Ethics Committee (SESREC) 3 (study number 10/S1103/63). An Advisory Committee comprising of four independent experts will monitor the study progress throughout the project.

### Identification and recruitment of participants

Recruitment of and liaison with individuals participating in the study will be carried out by community researchers. We will employ community researchers from the areas in which the study will be undertaken, who are familiar with the farming community and have detailed knowledge of the local area. The community researchers will assist with recruitment and will visit participants regularly, not only to collect information, but to maintain good relationships and to provide regular feedback on the progress of the study. It is hoped that such engagement will help minimise attrition of study participants. The following sections describe the general approaches that will be used for the recruitment of farmers and residents living near them into the study.

Local farms and orchards will be identified using publically available sources such as the Yellow Pages^2 ^as well as via the community researcher's knowledge of the local area. Farmers will be approached initially by a letter, briefly explaining the study aims and followed up via a telephone call. Telephone scripts and prompts will be used to assist with the telephone recruitment, with further details of the study aims and objectives, what input would be required from the farmer as well as questions to assess the farmers' suitability for participation in the study being asked.

A Microsoft Excel spreadsheet will be used to track recruitment. Each recruited farm will be assigned a unique identifier.

Eligible farmers are those who are likely to spray their crops with one or more of the pesticides under study (Table 1) and have residential areas within 100 m of these crops. Eligible farmers who have expressed an interest will be visited in person by the community researcher at a mutually convenient time to discuss the study in more detail and to discuss the process of data collection.

Once farmers are recruited, those households within approximately 100 m from the edge of a crop that is likely to be treated will be selected for recruitment.

Residents will be contacted firstly by letter which will also contain copies of two participation information leaflets, the first being for adults (defined as being 18 years and over) and the second for children aged 4-12 years. The participant information leaflet will be included at this stage to give recipients adequate time to consider whether they wish to participate. Leaflets for children will be piloted in advance for intelligibility and understanding amongst comparable non-sampled children. We will attempt to obtain a response via several methods. A reply form will be enclosed with the letter through which individuals can register their interest or otherwise in the project. In instances where we have a contact telephone number for the home, an approach will also be made via this communication route. However, in addition, as we anticipate difficulties in obtaining residents' phone numbers and that individuals may simply forget to return the form or contact the researcher, the letter will state that the community researcher will be working in the area during the week and that they will try to establish in-person contact.

As the success of this study will depend on enlisting and retaining volunteer participants, we will use a variety of ways to engage with the local community. This may include adverts in local newspapers and community notice boards and presentations to key groups in the community. In addition each individual participating in the study will be compensated for the time spent in completing questionnaires and providing urine samples.

Both adults and children in their care will be invited to participate. Children will, however, only be recruited alongside a consenting parent or guardian and consent must be given by the parent or guardian.

In addition, farmers and their partners and children (aged 4-12 years) will be given the opportunity to take part in the study by providing urine samples throughout the spraying season if they do not themselves spray or come into contact with the spray equipment or enter the crop following treatment with the pesticides. Where the farmer is the operator or the farmer states that he entered the field shortly after spraying, his results will not be considered. Similarly, farm workers who are actively involved in the application of pesticides will not be eligible for inclusion in the study.

A Microsoft Excel spreadsheet will be used to track residents' recruitment. Each household will be assigned a unique identifier. Once a household has agreed to participate in the study, unique sample identification numbers, based on the household identifier, will be allocated to each consenting participant.

Individuals who express an interest in participating in the study and who satisfy the inclusion criteria will then be visited in person by the community researcher at a mutually convenient time to discuss the study in more detail, explain the arrangements for data collection, and obtain written consent.

No excessive attempts will be made to obtain a response from residents or farmers. A response of 'no' to participation in the study will be accepted and no further contact will be made.

### Data collection

#### Farms

Participating farmers will be asked to sign a consent form confirming that they are willing to provide the study team with information concerning their pesticide usage. Participating farmers will be asked to provide information on planned pesticide usage for the forthcoming year. Farmers will also be asked to indicate what fields are theirs on a map of the area surrounding their farm and which, typically, have pesticides applied. In instances where farmers demonstrate that they already maintain comprehensive records of their pesticide usage, the researcher will ask whether copies of these can be made at regular intervals throughout the spray season. In any instances where detailed records are not already maintained, participating farmers will then be asked to record details of spraying events on a weekly basis throughout the spraying season using an adaptation of the form recommended in Annex F 'Code of practice for using plant protection products'.^4^

The community researcher will contact participating farmers on a regular basis to ensure continued participation, collect pesticide usage records and obtain updated information on their planned pesticide usage.

From the records of pesticide usage provided by the farmers we will obtain details, for example, of the pesticide used, date of application, method of spraying and time taken to spray. Details of prevailing weather conditions on the day of spraying and afterwards will be obtained from the relevant weather station from the Met Office. All of this information will then be entered into a designated Microsoft Access database at IOM's Edinburgh office.

At the last visit to the farm during a spraying season, the community researcher will recheck the farmers spray records to ensure that details of all relevant spray periods have been recorded. The participant will be issued with the appropriate value of gift vouchers and asked to complete and sign the compensation acknowledgement form to confirm receipt. Participants of the 2011 spray season will be asked if they would be happy for the community researcher to approach them for their involvement in the 2012 spray season and their response will be recorded and followed up as appropriate.

#### Residents

Interested individuals will be visited and the study explained to them and will be issued with further copies of the participant information leaflets. Informed consent will be obtained from those agreeing to take part and a short background questionnaire will then be administered. The questionnaire to be completed by adult participants includes questions concerning their personal details (e.g. age, gender, weight), lifestyle (e.g. leisure activities), occupational and para-occupational exposure to pesticides (exposure via, for example, family members, who work with pesticides and live in the same home) and pesticide usage within the home. The questionnaire to be completed by the adult on behalf of a child (4-12 years) participating in the study is shorter and includes questions concerning their personal details, education/nursery location and leisure activities.

Participating residents will be asked to provide a first-morning void urine sample (around 70 ml) on specified days during the spraying season. In addition, we will collect a limited number of urinary samples from participants outside the spraying season to determine background excretion of the biomarkers of interest.

Two strategies will be employed to collect urine samples throughout the spray season:

i) In instances where the farmer has indicated that that they are very likely to spray the pesticides of interest (Table 1) during a given period of the spray season, relevant participating households will be allocated one day of the week on which they are to provide weekly urine samples throughout the indicated period. Further, if we are made aware that spraying has occurred in an associated field involving one or more of the pesticides listed in Table 1 all relevant participating residents will be contacted and asked to provide an additional first-morning void sample on the day following the spray event.

ii) Where the farmer has indicated that it is more difficult to predict their usage of the pesticides listed in Table 1, and that such usage is unlikely, but good communication links have been established, participants will be asked to provide a urine sample preferably the day after each relevant spray event, although samples will be collected 2 days after a relevant spray event if the first day after is missed. For this strategy, the participants will be contacted by the community researcher when they have been made aware that the farmer intends to spray.

All participants will be supplied with sufficient sampling materials and will receive simple instructions for the collection of the urine samples. Along with each urine sample provided, we will ask the participants to complete a brief activity diary for the preceding two days.

The activity diary to be completed by adult participants requests information on time spent at home, time spent at home outdoors (e.g. in the garden), use of pesticides, and any potential occupational/para-occupational exposure to pesticides. In addition, the participants will be asked to provide information on consumption of any home grown vegetables. The questionnaire to be completed by the adult on behalf of a child participating in the study is shorter and includes questions concerning the time spent by the child in both outdoor and indoor environments.

Samples and diaries will be stored immediately after collection/completion in a cool bag by the participant. The community researcher will collect the cool bag from the participant's home on the designated day (within a period of no greater than 12 hours from sample provision), checking that it contains the expected number of completed study packs and that in each study pack, the sample code on the diary matches that on the urine sample receptacle. The community researcher will check that the diaries have been fully and correctly completed and that the date and time of collection has been written on the urine sample receptacle. In the event that any part of the questionnaire needs clarification the community researcher will approach the resident to rectify this.

All collected data will then be entered into a designated Microsoft Access database at IOM's Edinburgh office.

At the end of the data collection period participants will be asked if they would be happy for the community researcher to approach them for their involvement in the 2012 data collection period and their response will be recorded and followed up as appropriate.

#### Labelling and tracking samples

A robust sampling and tracking system will be implemented to ensure that the contextual information from the completed diaries remains linked to each collected urine sample and that the location of each urine sample is known. This will be achieved through the use of the unique farm, resident and sample identifiers along with the recording of all relevant information in Microsoft Excel spreadsheets and Access databases.

All study packs will be identified by a unique sample identification number which provide a permanent link to the individual which is known only to the project team. In instances where there is more than one participant within a household, a removable label will be placed on the sample pack, indicating which individual the pack relates to. Participants will be instructed to remove this upon collection of the urine sample and completion of the accompanying diary. This will ensure that the packs are used by the correct participants and also that their data are anonymised to those outside the study team. Participants will however be requested to record their date of birth on the urine samples receptacle and completed diary in order for the study team to verify that the correct study pack was used.

#### Urine sample storage and transportation

Upon collection by the community researchers the study packs (diary and urine sample) will be placed in a small table-top freezer. On a weekly to fortnightly basis (depending on the number of samples collected) the community researcher will courier the collected samples to the IOM, Edinburgh using next day delivery, or deliver them by hand (East Lothian only). The community researcher will ensure the frozen study packs are securely packed in the packaging material supplied by IOM. Upon receipt at the IOM each diary and urine sample will be checked, logged, with the urine sample again being stored in a freezer and the questionnaire being separately stored in a secure tambour.

Selected frozen samples will then be couriered, using next day delivery, to HSL, Buxton and an electronic record of the IDs of the samples couriered will be sent to HSL. The urine samples will be securely packed. Upon receipt of the samples, the sample numbers will be checked against the electronic copy to ensure that the correct samples have been received. The samples will then be stored following HSL's in-house procedures.

Freezer temperatures, including the table-top freezers, will be maintained within the range of -15 to -20°C and the temperature will be logged regularly.

### Urine sample analysis

#### Selection of samples for analysis

Not all collected urine samples will be selected for analysis, with those samples fulfilling one of the following three criteria being selected:

1. Urine samples collected within 2 days after relevant spraying events

2. Up to 3 samples within the spraying season (randomly selected) to allow background pesticide metabolite levels within the spray season to be determined.

3. Up to 3 samples collected outside the spraying season to allow background pesticide metabolite levels outside the spray season to be determined.

Urine samples collected within 2 days after a relevant spraying event will be analysed for the active ingredients listed in Table 1 relevant to the given spray event. Background samples, both within and outside the spray season, will be analysed for all relevant active ingredients for which urine samples have been collected for spray events.

#### Laboratory analysis

Urine samples will be analysed for the relevant pesticide metabolites using gas or liquid chromatography/mass spectrometry (GC/MS or LC/MS) by HSL. The laboratory will follow ISO9001 record keeping and other relevant quality procedures. Metabolite concentrations will be expressed either as μg/l or corrected for creatinine concentration.

All samples and results will be logged into HSL's Biological Monitoring Database. Samples will be identified by the anonymised sample identification number; HSL will not receive any personalised data.

The results of the urine sample analysis will be reported, electronically, by sample ID number to IOM for data analysis.

### Reporting and participant feedback

Participants will be given regular updates on the progress of the project, through newsletters, individual letters and direct feedback from the community researchers. Regular updates of the projects progress will be posted on the project specific website (URL: http://www.pesticidebiomonitoring.org).

It is not intended to provide participants with details of their individual urinary results for of the following reasons:

• Not all collected urine samples will be analysed;

• We will only select urine samples for analysis which coincide with spray events (as reported by the farmers) as well as randomly selected urine samples within and outside the spray season to obtain information on background exposures;

• Only specific pesticide metabolites will be analysed for; and

• We can only interpret the results in terms of exposure, not possible ill-health effects.

Participants will however, upon request, be forwarded a summary of the overall urinary metabolite results and the study report.

### Modelling and statistical analysis

#### Prediction of residential exposure

We will use a simple pharmacokinetic (PK) model based on that developed by Rigas et al. (2001) [8] to convert measured urinary metabolite levels into systemic exposure levels and to predict urinary metabolite levels obtained using the regulatory risk assessment procedures and the BREAM model [6]. We have previously used this model to predict the levels of metabolites from cypermethrin (3-PBA, DCVA), mancozeb (ETU) and chlorpyrifos (TCPy) [1]. The model assumes that the pesticide is absorbed into a single body compartment and that the distribution of the pesticide, or its metabolite, in the body is approximated by a volume of distribution. This model describes the pathways from uptake to absorbed dose, to metabolite concentration in tissues and, finally, to urinary excretion. Information on other potential sources of exposure will be obtained from the questionnaires completed when the samples are provided and taken into account.

The PK model allows for uncertainties in the model parameters by using probabilistic modelling techniques. As in our previous study [1], we propose to use probabilistic methods to investigate the relative effects of potential sources of uncertainty on the calculation of the urinary metabolite levels. The probabilistic methods allow the model inputs or parameters to vary according to some specified distribution, rather than using specific point estimates. The distributions will be derived from knowledge of the variability of the data taken from existing literature and from observations during the study. The models will be run several thousand times where, for each run, a value of each of the model parameters for which there is uncertainty will be probabilistically selected from the specified distributions and the resulting metabolite levels will be estimated. This will result in an estimate of the distribution of the predicted urinary metabolite level, rather than just a point estimate.

Analyses will be carried out to determine the sensitivity of the predicted systemic dose and urinary metabolites to variation in the model input parameters.

The principal statistical comparison will be between the distribution of predicted urinary metabolite levels calculated from the regulatory models and the biomonitoring data within this study.

#### Statistical analyses

The following statistical analyses will be carried out using the urinary metabolite data:

1) We expect that a relatively large percentage of the background samples during the spraying season and the samples collected outside the spraying season will be below the limit of detection. A median urinary level for TCPy of 5.3 μg/l in repeated samples collected during a longitudinal study from 80 individuals in Maryland, with approximately 20% of samples below the limit of detection, has been reported [9]. Meeker et al. (2005) found a geometric mean of 2.3 μg/l in urine of 360 men, with only 7% of samples being non-detects, but the limit of detection of the analytical technique was very low (0.25 μg/l) [10]. A UK general population study showed 'non-detect' rates of 15% for 3-PBA, 37% for DCVA and 54% for ETU [7]. These are therefore the maximum 'non-detect' rates expected in our residents however this will be checked following the 2011 data collection.

We aim to use a multiple-imputation technique to replace the values below the limit of detection [11].

2) The long-term exposure during the spraying season will be estimated directly using the results from the urinary analyses. This will provide an overestimate of the actual long-term exposure, as this includes only up to 3 background samples for days when no pesticide spraying took place. We will also aim to estimate a more accurate long-term exposure during the spraying season by imputing urinary results for all the non-spraying days, based on the background measurements.

3) Comparison of the long-term systemic exposure during and outside the spraying season and analyses of the within and between-individual variability in the urinary metabolite data.

4) Explore factors that may explain differences in exposure levels between communities and regions and between spraying and non-spraying periods. To determine trends in urinary metabolite levels and assess whether the levels following spraying events are higher than background levels we will use hierarchical multivariate analyses, including both random and fixed variables. Random variables will include community (participating farm with surrounding residents) and individuals. Fixed variables will include environmental factors such as weather conditions and geographical details; spraying information obtained from the farmer (such as spraying technique, quantity used, etc.); information from the resident questionnaire (such as percentage of time spent at home and outside); and demographic details (age, gender, etc.).

5) The long-term estimate of systemic exposure levels based on the results of the urinary metabolite measurements, converted using the PK model, will be compared with the predicted levels based on the regulatory risk assessment process and the BREAM model. The aim of these analyses will be to determine whether the outcomes from the regulatory risk assessment are sufficiently conservative.

### Power

In order to obtain an estimate of the number of subjects required for the study a range of conservative power calculations were carried out for a number of pesticides identified as being most likely to be applied during the spray seasons in the target areas. These power calculations were based on collecting urine samples from 30 individuals with two replications per person and a range of estimated standard deviations (SD). Information about geometric mean (GM) and geometric standard deviation (GSD) background levels were obtained from various sources [7,12-15]. Where only background levels were available, the power calculations were based on an ability to detect a doubling of background levels, with the GSDs ranging between the GM levels.

Rather than reproducing all the power calculations we provide one example for background levels for trans-DCVA, a metabolite of cypermethrin. Estimated background mean and treatment mean urinary concentrations of 0.7 and 1.4 μg/l on log-scale were obtained from background levels [13], which gave an estimated difference of 0.7 μg/l. Power calculations were carried out for SD of the mean difference ranging from 0.6 to 1.4 μg/l, in steps of 0.1 μg/l, using G-Power statistical software for power calculations [16], with the results presented in Table 2.

**Table 2:** Sample size required to attain 95% and 75% power to detect a difference of 0.7

	Sample size required	
Standard deviation on log-scale	95% Power	75% Power
0.6	12	8
0.7	16	10
0.8	20	12
0.9	24	14
1.0	29	17
1.1	35	20
1.2	41	23
1.3	47	26
1.4	54	30

**Figure 1:**
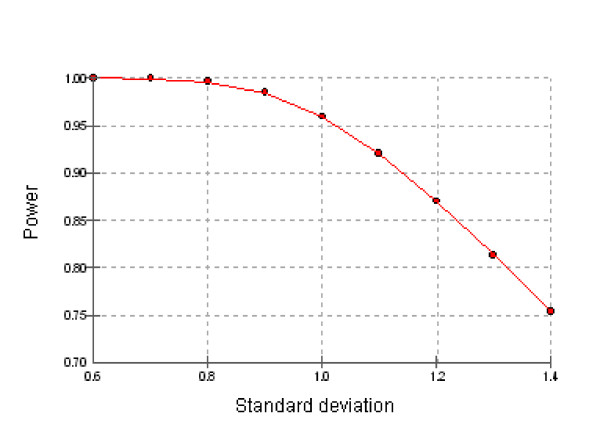
**Statistical power associated with a range of standard deviations for a sample size of 30**.

Statistical power associated with a range of standard deviations for a sample size of 30 is shown graphically in Figure 1.

Overall, the calculations showed sufficient power to detect a doubling in mean of specific analytes (power generally in excess of 75% depending on pesticide metabolite investigated). The estimated power for 3-PBA, a generic metabolite of pyrethroids, was lower, ranging between 38 and 74% depending on the (random) variability of urinary levels.

It must be noted that the power calculations are conservative estimates, since in the study we will have multiple replications per person and so we consider the power calculations show that 30 individuals should be sufficient to detect a change in analyte level of the anticipated magnitude and that in many cases, 20 individuals should be sufficient. However, it is intended to repeat the power calculations when information from the first year of the study is available and, if necessary, the numbers to be recruited will be revised.

## Discussion

This study protocol was implemented for the 2011 sample and data collection procedures. Based on experience to date, no major changes to the protocol are anticipated for the 2012 spray season although the pesticides and regional areas for inclusion in 2012 are still to be confirmed.

## Competing interests

The authors declare that they have no competing interests.

## Authors' contributions

KG is the project leader, the lead author responsible for the drafting and completion of the research protocol and also for the drafting of this manuscript. MvT is the Principal Investigator of the study. AS and MvT were responsible for developing the original project proposal. AS, JC, KJ and JC were responsible for identifying the research question. JC, KJ and JC contributed to the development of the study protocol. LM and PT contributed to the development of the protocol and study design as members of the research team. All authors have provided comments on the drafts and have read and approved the final version of this manuscript.

## Endnotes

^1 ^http://www.pesticides.gov.uk/approvals.asp?id=2427

^2 ^http://www.yell.com

^3 ^http://www.pesticides.gov.uk/Resources/CRD/Migrated-Resources/Documents/C/Code_of_Practice_for_using_Plant_Protection_Products_-_Complete20Code.pdf

## Pre-publication history

The pre-publication history for this paper can be accessed here:

http://www.biomedcentral.com/1471-2458/11/856/prepub
